# On the historical roots of creationism and intelligent design: German Allmacht and Darwinian evolution in context

**DOI:** 10.1007/s12064-021-00341-x

**Published:** 2021-03-24

**Authors:** Elizabeth Watts, Ulrich Kutschera

**Affiliations:** Biology Education Research Group, Friedrich-Schiller-Universität Jena, Am Steiger 3, Bienenhaus, 07743 Jena, Germany

**Keywords:** Creationism, Creation science, Intelligent design, Science education, Vernon Kellogg

## Abstract

As detailed in a Letter published in *Science* in 2017, the adherents of creationism and intelligent design are still active in promoting their biblical-literalist views of the origin and evolution of life on Earth. In this contribution, we take a look at this ideological phenomenon in the USA and analyze the philosophical roots of this ongoing movement. Specifically, we discuss Vernon Kellogg’s book entitled *Headquarters Nights* (1917) with reference to the German ‘Allmacht’ (English—omnipotence) and Darwinian evolution to demonstrate how this publication bolstered the development of active anti-evolutionism in the USA among American fundamentalist Christians, inclusive of the Intelligent Design (ID)-agenda. The current activities of creationist associations in the USA and Germany are summarized, with reference to a new pro-ID-group established in Austria in 2019 that is sponsored by the Discovery Institute in Seattle, Washington (USA).

## Introduction

In September 2017, the journal *Science* published a Letter entitled “Intelligent Design endangers education” summarizing the current status of science education in the USA (and other countries such as Brazil and Turkey) with reference to biology and geology (Silvia 2017). It concludes that despite efforts to combat religious interventions in biology classes, “proponents of creationism and the Intelligent Design (ID)-hypothesis continue to permit to teach creationism alongside evolution” (Silva 2017, p. 880). Although it is well known that biblical literalism, a major component of the creationist movement, originated in the USA, the philosophical roots of this “anti-science-agenda” are largely unexplored. In the present article, which was motivated by the “wake-up call” of Silva (2017), we take an in-depth look at the roots of this movement with a special focus on the influential book *Headquarters of Nights* authored by American evolutionary biologist Vernon Lyman Kellogg (1867–1937). This book was published in 1917—exactly one hundred years prior to Silva’s publication—highlighting the length and tenacity of this issue in the USA.

In order to better understand the momentum behind this movement, we have undertaken a historical analysis of the philosophical roots of creationism in America. In this article, we will address the religious basis of creationism as the logical by-product of increasing evangelicalism and biblical literalism. Furthermore, we discuss why America was a particularly fitting location for the development of this religious movement. Once we have addressed the religious and cultural basis of creationism, we then take a specific look at how the expansion of public schooling, the introduction of evolution to the textbooks, the role of science in World War I began to cause apprehension among American evangelicals and how this turned into a vigorous anti-evolution movement with the publication of *Headquarters Nights* when Kellogg drew the connection between German war atrocities and Darwin’s theory of evolution. Finally, we discuss a new US-sponsored Intelligent Design (ID)-agenda in Europe that was established recently (January 2019) and address the 40th Anniversary of the “word and knowledge” (Wort und Wissen)-society in Germany.

## Christian fundamentalism and biblical literalism

In order to understand the general sense of apprehension surrounding the teaching of evolution, it is important to understand two main points: (1) the prevalence of evangelicalism in America at that time and (2) how evangelical Christianity differs from mainline Christianity. Evangelicalism is a transdenominational movement within Protestantism that upholds the belief that the Bible consists of the doctrine of salvation, which can be attained by giving your life to Jesus—other central components include the “born again” experience in receiving salvation, an emphasis on the authority of the Bible as God’s direct communication to humanity, and a felt urgency in the need to “share the good news” and bring others to Jesus (Stanley 2013). According to historian David Bebbington, the four key aspects of evangelicalism can be summed up as: conversionism, biblicism, crucicentrism, and activism (1993). Fundamentalists Christians can be understood as a subset of evangelicals who also maintain a literalist view of the Bible. The term fundamentalist came into prevalence in the early twentieth century.

**Fig. 1:**
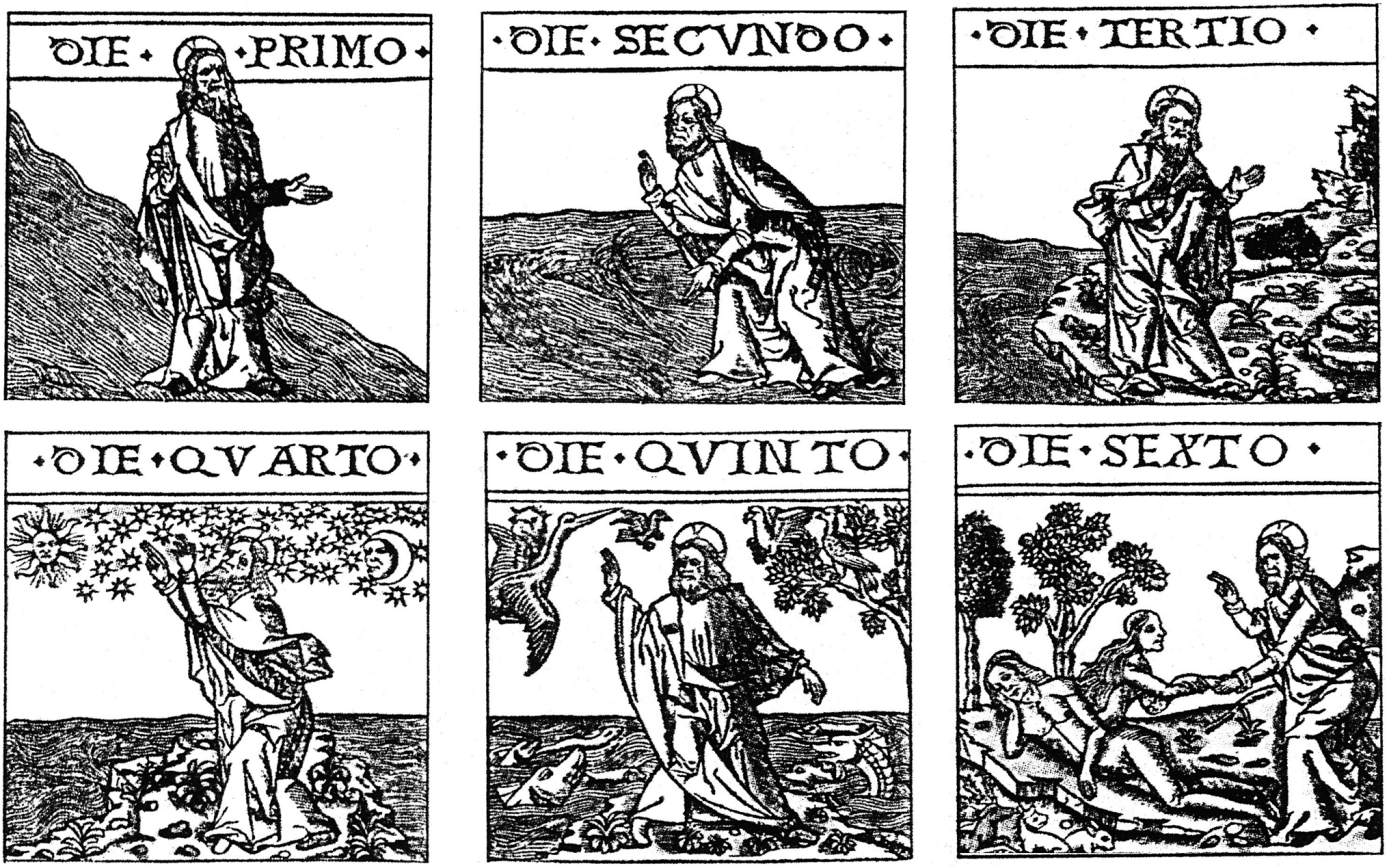
The creation of Life on Earth according to the Biblical account, as detailed in the book of Genesis (woodcut, ca. 1800)

According to Hood et al. (2015), the emphasis on biblicism and the idea of an inerrant Bible are the key defining points between fundamentalist and traditional religious adherents, “What distinguishes fundamentalism from other religious profiles is its particular approach toward understanding religion, which elevates the role of the sacred text to a position of supreme authority and subordinates all other potential sources of knowledge and meaning” (Hood et al. 2015, p. 13). The traditional theological approach to religious practice varies greatly from this fundamentalist approach as the Bible is not only considered as a source of religious inspiration, but also seen as an infallible document that contains all of the *how, what,* and *when* the living world came to be (Fig. 1).

The term ‘creationist’ can be used to describe these fundamentalist circles starting in the 1920s. However, while many current creationists refer to themselves as evangelicals rather than fundamentalists, we will continue to use the term fundamentalist to highlight the differentiation between evangelicals who uphold literalists beliefs (fundamentalists) and those who do not. It was during this time that fundamentalists took on an official contrarian position to evolution in response to the inclusion of evolution in textbooks in ever expanding public schools. The creationists took the evangelical emphasis on biblical literalism to a higher level and became convinced of an irreparable contradiction between evolution and Genesis, thus requiring an utter rejection of evolution in order to preserve their relationship with the biblical God and maintain their rightful position in heaven (Ham 2012). The falsehood of this claim has been proven not only through evolutionary biologists who have maintained their faith as exemplified by leading evolutionist Theodosius Dobzhansky (1900–1975) (Kutschera 2006) but also through theologians who have made major contributions to science, such as the monk Gregor J. Mendel (1822–1884) or Catholic priest Georges Lemaître (1894–1966).

Due to the focus on activism and conversionism within evangelical Christianity, many fundamentalists began to push for evolution to be banned from the classroom, in order to have supernatural explanations for the origin of man, earth and the universe included in the lesson plans to replace or offset the naturalistic explanations already present in public school science education (Scott 2009). It has been clear to historians for many years that the creationist phenomenon originated in the USA and here we examine why the USA offered such fertile grounds for the emergence of this strain of fundamentalist Christianity. In addition, we address how this is related to the idea of American exceptionalism, i.e., the postulate that the USA is unique and differs from all other nations.

## Historical roots of creationist ideology in early American exceptionalism

According to journalist Ian Tyrell, no one has done more to promote the idea of American exceptionalism than former US President Ronald Reagan (1911–2004) as “Reagan promoted the image of the USA as a shining ‘city upon a hill’” (Tyrell 2016). This mention of a shining city on a hill is actually in reference to a sermon held by John Winthrop (1588–1649) in 1630 as part of the voyage of the Winthrop fleet which encompassed eleven ships and 1000 Puritans on their way to settling a new country. This sermon, which was based on Jesus’ Sermon on the Mount in Matthew 5:14, “You are the light of the world. A city that is set on a hill cannot be hidden,” exemplified the Puritans’ belief that they were a chosen people who were there to create a Christian nation. This concept was also reiterated in early America in the Quaker idea of the “holy experiment” (Barbour and Frost, 1998).

This idea of American exceptionalism continued throughout the development of the USA, yet at the start of the nineteenth century, a fear began to arise among the American people that their new nation may not become the strong Christian society that they had envisioned (Watts et al. 2016a, b). Many in fact believed that America was in moral danger as fewer and fewer Americans were visiting mainline churches in the early 1800s, due to the westward movement to the frontier. While the new western frontier offered many new opportunities for settlers, it was initially lack of formal schooling or churches. Within this void of formal churches, fairly uneducated settlers began to read the Bible at face value, seeing this “Holy Book” as their direct line to God. At the same time, traditional church masses were replaced by a new form of congregation known as “revivals.” During these “revivals,” which simply took place in the woods and attracted large masses, charismatic ministers warned the masses of the spiritual crisis of that the new nation was in. There was a sense of urgency to bring people to Christ in order to save the citizens and the nation (Watts 2018).

As a result, the number of revivals increased exponentially and by 1811, more than 1,000,000 Americans were visiting at least one religious revival per year. By the mid-nineteenth century, the religious landscape of the USA had been completely transformed from mainline Christianity to an emotional and personal strain of Christianity (Belton 2012). It was during this same time that multiple religious sects began popping up in the USA, such as the Shakers, the Mormons, the Jehovah Witnesses, the Seventh-Day Adventists, the “Christian Scientists.” The northeastern part of America during this time was such a hotbed of revival that upstate New York was dubbed the “burned-over district,” referring to the fact that evangelists had exhausted the region’s supply of unconverted people (Cross 1950).

**Fig. 2:**
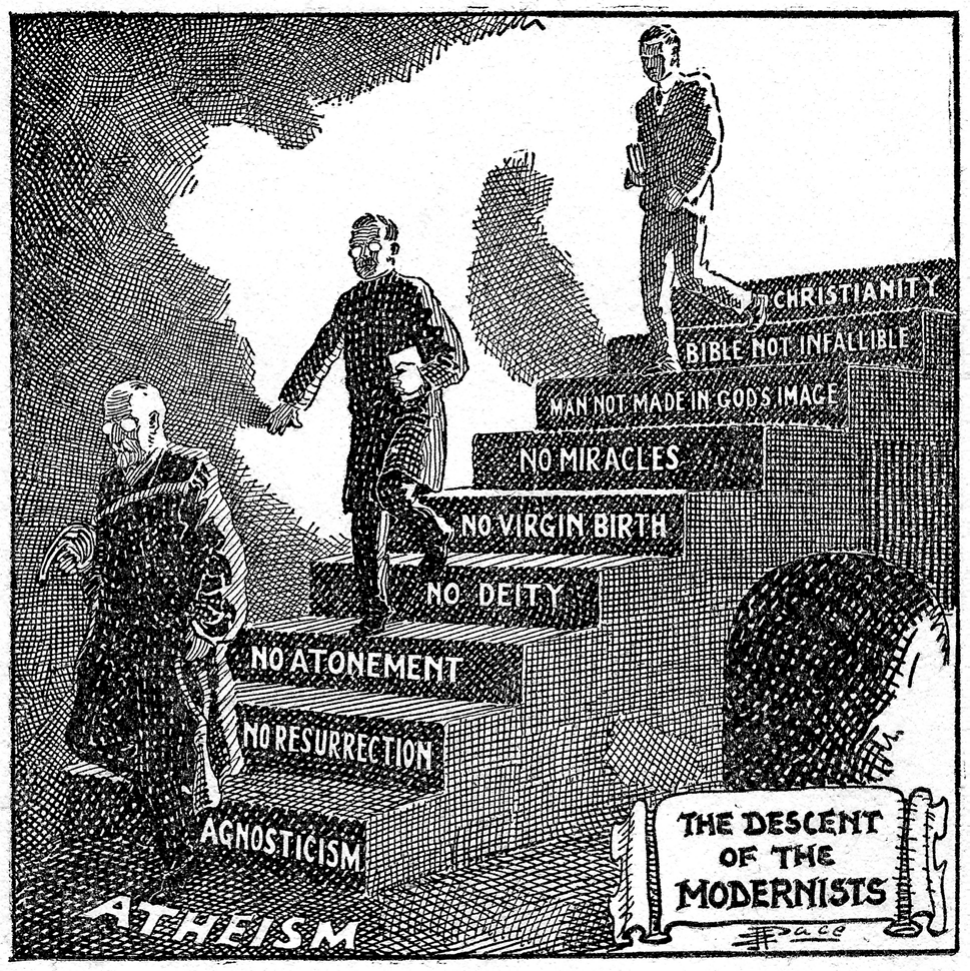
Cartoon illustrating the “Descent of the Modernists,” E. J. Pace, Christian Cartoons, 1922

So, throughout the history of the USA, there has been an idea that Americans have a special and personal relationship with God. John Winthrop took this idea of a city on the hill from the Gospel of Matthew and over time this Biblical image became personally American. Since Christianity postulated that humans were the crown of creation, evangelical ministers and politicians throughout American history claimed that Americans not only represented the “top species” of creation, but God’s chosen people. As a result, a loss of Christian faith was interpreted as a “descent to atheism” (Fig. 2).

## The impact of Darwin’s theory on the minds of young Americans

This idea that humans were seated at the crown of creation (and that Americans were the crown of the human pyramid) went unchallenged for generations until Charles Darwin’s (1809–1882) theory of organismic evolution was published in 1859. The general population became exposed to Darwin’s new ideas when his theory of descent with modification was introduced to American high school textbooks in 1914 (almost 50 years after the publication of *Origins*). The inclusion of Darwin’s ideas in Hunter’s 1914*Civic Biology* coincided with a general educational reform in the USA that greatly subsequently increased the impact of this textbook addition (Shapiro 2013). While only 6% of American seventeen-year-olds were attending school at the turn of the century, 50% of Americans were high school graduates by 1940 (Herbst 1996). This rapid expansion of public schools and subsequent increase in attendance to these state-supported institutions meant that masses of young American minds were exposed to the idea of evolution (Slawson 2005). From the pages in Hunter’s *Civic Biology*, they learned the following:

*Man’s Place in Nature*.—Although we know that man is separated mentally by a wide gap from all other animals, in our study of physiology we must ask where we are to place man. If we attempt to classify man, we see at once he must be placed with the vertebrate animals because of his possession of a vertebral column. Evidently, too, he is a mammal, because the young are nourished by milk secreted by the mother and because his body has at least a partial covering of hair. Anatomically we find that we must place man with the apelike mammals, because of those numerous points of structural likeness. The group of mammals which includes the monkey, apes, and man we call the primates (Hunter 1914, p. 195)

For many American, particularly fundamentalist evangelicals, this idea did not sit well, as evolution was perceived as being inconsistent with the literal idea of special creation (Kutschera 2009a, b, 2017; Larson 1997; Watts 2018). Moreover, it has been suggested that the crux of the problem initially was not only that Darwin’s naturalistic theory of evolution contradicted the supernatural biblical account of creation, but that it also conflicted with a deep-rooted belief regarding Americans’ special relationship with God, as Hasia R. Diner (born 1946) describes it: “A core religious belief was that human beings were the crown of creation. And in very American terms, the American was also the crown of creation. But now, reading these accounts of Darwin, one couldn't say that any longer. Darwinism undermined the notion of what it means to be an American” (Belton 2012).

## Vernon Kellogg and the German ‘Allmacht’

Critical views of evolution and a sense of danger associated with the theory gained ground as Vernon Lyman Kellogg (1867–1937) drew a clear link between German war atrocities and neo-Darwinism in his 1917 publication *Headquarters Nights*.

**Fig. 3:**
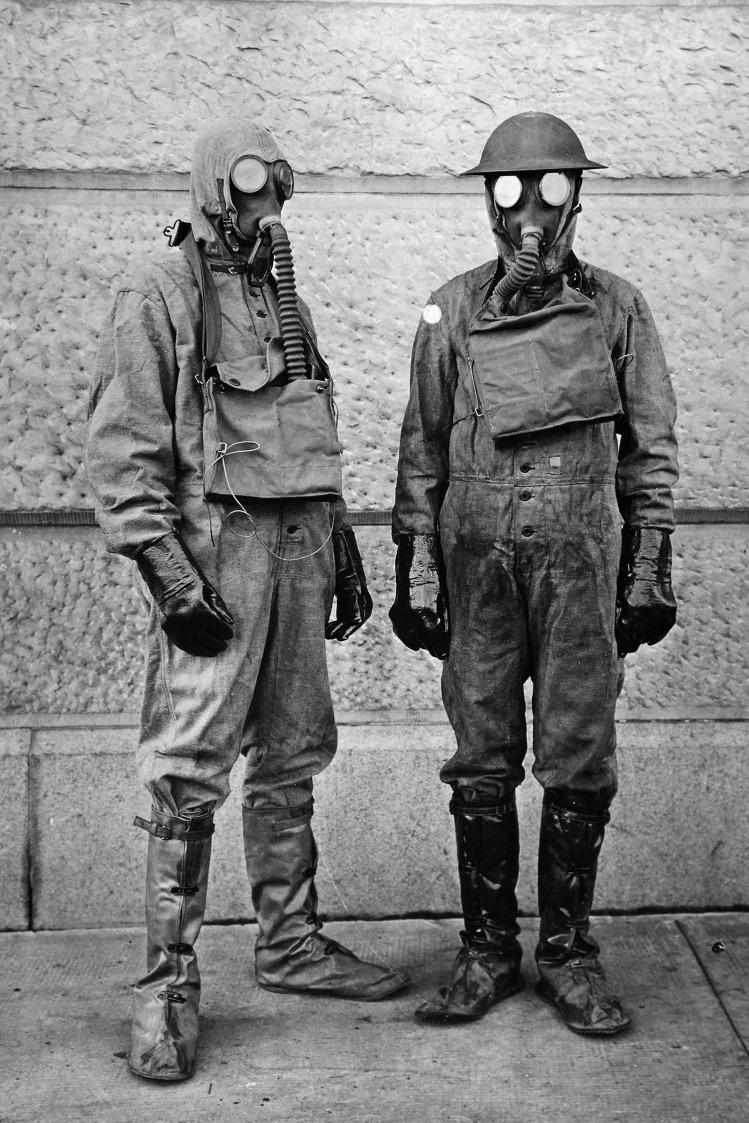
Protective suits worn by American scientists during the development of new chemical weaponry. (Photo: National Archives and Records Administration)

Although the USA’s involvement in the war was relatively brief—entering WWI in April 1917—the war affected the American’s view of science in general and evolution as WWI was the first modern war where countries actively attempted to apply modern scientific knowledge to perfecting warfare. Even prior to the USA’s involvement in the war, the *National Academy of Sciences* had anticipated the need for collaboration between scientists and the military (Fig. 3). To address this need, the *National Research Council* was established in 1916 by President Woodrow Wilson and once the NPC had been established, the National Academy’s foreign secretary, George E. Hale (1868–1938), sent a message to his counterparts in Britain, France, Italy and Russia reading “The entrance of the United States into the war unites our men of science with yours in a common cause.^1^”

The use of modern technology and science was seen as a necessary means of competing against the Germans who first used chlorine gas on the battlefield in 1915, causing international outrage. When the USA first entered the war, they were unprepared for this new warfare style and recognized their own need for gas troops, who were then deployed in 1917.^2^ This new brand of scientific warfare resulted in an unprecedented loss of human life. For conservative evangelicals, these horrendous losses exacerbated their general fear of modernism and specific trepidation regarding science as Randall M. Miller describes, “From the traditionalist point of view, this war was a demonstration of all that had gone wrong, and a warning because God, they believed, gives warnings. He visits his wrath upon the unrepentant people. The world seemed to be coming apart (Miller 2012).” While all of the involved countries had ultimately participated in the grievous losses, the Germans became equated with evil during this time as publications claimed that the German military forces had poisoned French wells and children’s candy (Humes 2007).

**Fig. 4:**
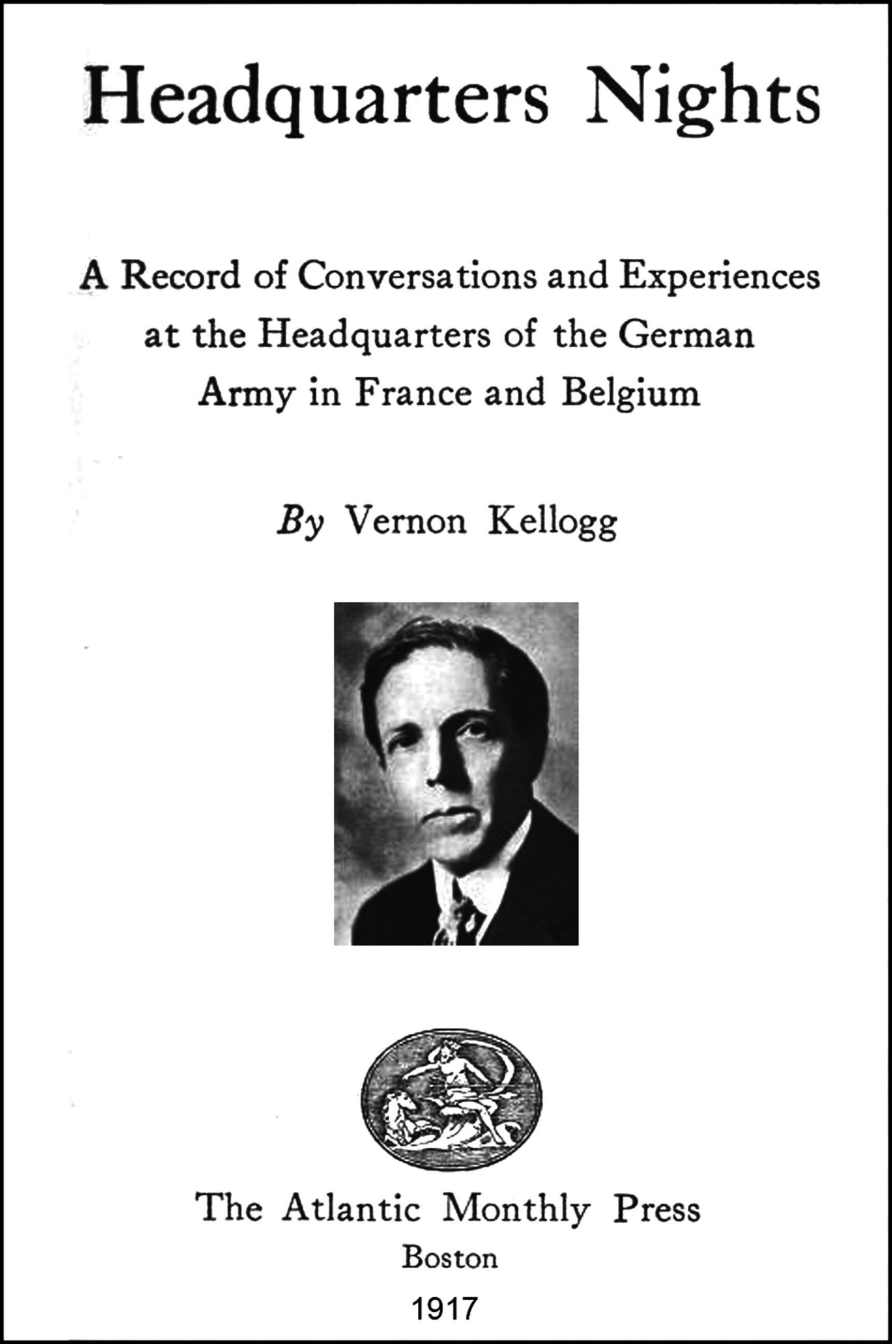
Title page, with a portrait of the author, of Vernon Kellogg’s *Headquarters Nights*, published in 1917 in the USA

Understanding the new role of science in this war and the American’s perception of the war is relevant when weighing the impact of Kellogg’s 1917 publication. Kellogg was an American entomologist and evolutionary biologist, who was a professor of entomology at Stanford University 1894 to 1920. He had a two-year hiatus during this period (1915 and 1916) when he served as the director of Hoover’s humanitarian American Commission for Relief in Brussels, Belgium. While in Brussels he often dined with the officers of the German Supreme Command and he later published an account of these conversations in his 1917 book *Headquarter Nights*. In his book, he described his shock at the social Darwinist motivations used by the Germans to defend their wartime actions, writing, “the creed of survival of the fittest based on violent and fatal competitive struggle is the Gospel of the German intellectuals (1917, p. 28)” (Fig. 4).

Kellogg understood the importance and centrality of evolutionary theory within the sciences and believed that all “researchers needed to incorporate evolutionary theory into all aspects of biological research” (Largent 1999, p. 466). In fact, Kellogg’s views on evolution were very similar to his contemporary, zoologist and geneticist Ludwig Hermann Plate (1862–1937), who was a pupil and successor of Ernst Haeckel (1834–1919) and campaigned for a revival of the “old Darwinism” (Hoßfeld and Levit 2011; Hoßfeld et al. 2019; Levit and Hoßfeld 2006, 2019; Watts et al. 2019). Plate combined selectionism with neo-Lamarckian ideas and orthogenesis and was seen by many contemporaries worldwide as a proper advocate of Darwinism (Hoßfeld and Levit 2011; Levit and Hoßfeld 2006). Kellogg cited Plate profusely and his own view of the origin of species was a composite of Darwinism, orthogenesis, mutation, Lamarckian inheritance and other unknown factors (Dean 1908).

Kellogg’s scientific beliefs regarding evolutionary theory and the relative importance of evolutionary mechanisms are important to understand because he was able to draw a link between the neo-Darwinistic focus on *natural selection* and the resulting *social-Darwinistic* principles applied by the German *Allmacht*, in order to emphasize their *incorrect* understanding and application of evolutionary theory. However, the general public, who did not understand the intricacies of the scientific debate surrounding evolutionary theory during this time, simply understood Kellogg’s criticism as a connection between general *evolutionary theory* and *German militarism*. Ironically, Kellogg himself was painfully aware of how difficult it was to keep up with the ever changing advancements in evolutionary science even for educated readers as he wrote in the forward to *Darwinism To-day*: “Both destructive criticism of old, and synthesis of new hypotheses and theories, are being so energetically carried forward that the scientific layman and educated reader, if he stand but ever so little outside of the actual working ranks of biology, is likely to lose his orientation as to the trend of evolutionary advance” (Kellogg 1907, p. iii).

In looking at Kellogg’s book *Headquarters Nights,* we see how he described natural selection as the creed of the *Allmacht* and how the Germans had (mis)used Darwin’s ideas in order to justify their cruel actions throughout the war: “Well, I say it dispassionately but with conviction: if I understand theirs, it is a point of view that will never allow any land or people controlled by it to exist peacefully by the side of a people governed by our point of view. For their point of view does not permit of a live-and-let-live kind of carrying on. It is a point of view that justifies itself by a whole-hearted acceptance of the worst of Neo-Darwinism, the Allmacht of natural selection applied rigorously to human life and society and Kultur” (Kellogg 1917, p. 22). It should be noted that the term “Allmacht der Naturzüchtung” was coined by the German zoologist and evolutionary biologist August Weismann (1834–1914) in defense of the “Darwin-Wallace-Principle” of natural selection (Weismann 1893).

In the quote given above, we see that Kellogg emphasized the alleged use of *neo-darwinistic* principles by the Germans. From an evolutionary biologist standpoint, he was making an important distinction between a neo-darwinistic focus on natural selection over all other evolutionary mechanisms such as symbiosis, mutualism, and orthogenesis. While Neo-Darwinism places all focus on the struggle to survive as the sole means of evolution, Kellogg argued in his 1907 book *Darwinism To-day* that natural selection alone leads to constancy, not variability and that the changes driven by natural selection are quantitative in nature, not qualitative (Kellogg 1907). In *Headquarters Nights*, Kellogg also argued his view of the falsehood of focusing on natural selection alone as he pointed out the power of the mutual-aid principle over the mutual-fight principle:

Again, the adoption by two widely distinct and perhaps antagonistic species of a commensal or symbiotic life, based on the mutual-aid principle—thousands of such cases are familiar to naturalists—would ameliorate or abolish the interspecific struggle between these two species. Even more effective in the modification of the influence due to a bitter struggle for existence, is the adoption by a species of an altruistic or communistic mode of existence so far as its own individuals are concerned. This, of course, would largely ameliorate for that species the intra-specific phase of its struggle for life. Such animal altruism, and the biological success of the species exhibiting it, is familiarly exemplified by the social insects (ants, bees, and wasps). As a matter of fact, this reliance by animal kinds for success in the world upon a more or less extreme adoption of the mutual-aid principle, as contrasted with the mutual-fight principle, is much more widely spread among the lower animals than familiarly recognized, while in the case of man, it has been the greatest single factor in the achievement of his proud biological position as king of living creatures (Kellogg 1917, p. 26- 27).

The term “mutual-aid” in the context of evolutionary theory was coined by Russian geographer, philosopher, and naturalist Peter Kropotkin (1842–1921) in his 1902 book *Mutual Aid: A Factor of Evolution*. In this collection of essays, Kropotkin discusses the role of mutually-beneficial cooperation and reciprocity (or “mutual aid”) in the animal kingdom and human societies as an argument against theories of social Darwinism that emphasize competition and survival of the fittest. Although Kellogg refers to mutual aid in *Headquarters Nights* without citing Kropotkin, he did cite Kropotkin in his—zoology textbook *The Animals and Man*, which contained an entire chapter devoted to the discussion of mutual aid and communal life. Kellogg’s reference to mutual aid shows that his interpretation of evolution was arguably wider or more inclusive than even Plate’s.

For Kellogg, it was clear that the idea of natural selection described a struggle between an organism and its environment and not between different organisms (Ruse 2018) as seen in the excerpt above. It appears that Kellogg’s intent in *Headquarters Nights* was to argue that the narrow definition of evolution offered by neo-Darwinism may have harmful consequences not only for evolutionary theory, but also for social life.

The general public who read *Headquarters Nights* did not however understand that Kellogg was addressing a complex scientific debate regarding the true mechanism of evolution and the proper understanding of ‘the struggle for survival.’ The fine details of his argument were lost on the majority of readers and instead of understanding that Kellogg was opposed to the narrow understanding of evolution according to neo-Darwinistic principles, the book appeared to be a general criticism of evolution as the basis for German militarism.

Due to the extreme poor image of the Germans during this time, i.e., one associated with brutality and war crimes, Kellogg's observations and the connection that he made between the German militarism and Darwin had a particularly powerful effect. This effect was expounded by the fact that President Theodore Roosevelt (1858–1919) wrote the foreword of the book, stating: “One of the most graphic pictures of the German attitude, the attitude which has rendered this war inevitable, is contained in Vernon Kellogg’s ‘Headquarters Nights.’ It is convincing, and an evidently truthful exposition of the shocking, the unspeakable dreadful moral and intellectual perversion of character which makes Germany at present a menace to the whole civilized world. The man who reads Kellogg’s sketch and yet fails to see why we are at war, and why we must accept no peace save that of overwhelming victory, is neither a good American nor a true lover of mankind” (Roosevelt in Kellogg 1917, p. 13). Thus, Kellogg (1917) drew the connection between neo-Darwinistic (-Weismannistic) thinking and evil, while Roosevelt made a link between patriotism and rejection of evolution.

Kellogg’s book had a particularly profound effect on William Jennings Bryan (1860–1925), whose already cynical view of evolution was fueled by *Headquarters Nights* (Gould 1977). Bryan had already vocalized his concerns regarding evolution, warning Americans in a 1909 lecture that Darwin’s theory could undermine the foundations of morality, “The Darwinian theory represents man as reaching his present perfection by the operation of the law of hate—the merciless law by which the strong crowd out and kill off the weak. If this is the law of our development then, if there is any logic that can bind the human mind, we shall turn back toward the beast in proportion as we substitute the law of love (Bryan 1909, pp. 15–16).”

Following the reading of Kellogg, Bryan began touring the USA in the 1920s, becoming one of the most prominent religious figures in the country (Kazin 2006). Evangelicals rallied around him also appropriating the belief in the link between evolution and the darkest evils of mankind. Again, it is clear that Bryan also misunderstood Kellogg’s true intention. Kellogg did not say that evolution was the creed of the *Allmacht*, he said *natural selection* was the creed of the *Allmacht* and clearly pointed out that the Germans had omitted to understand many other potential mechanisms of evolution, “Altruism or mutual aid, as the biologist—prefer to call it, to escape the implication of assuming too much consciousness in it—is just as truly a fundamental biologic factor of evolution as is the cruel, strictly self-regarding, exterminating kind of struggle for existence with which the Neo-Darwinists^3^ try to fill our eyes and ears, to the exclusion of the recognition of all other factors (Kellogg 1917, pp. 27–28).”

Despite Kellogg’s actual intention of pointing out how the German *Allmacht* had misunderstood and misused evolutionary theory, his publication helped ignite a crusade against the teaching of evolution under the leadership of Bryan. In the context of this crusade, conservative evangelicals began to refer to themselves as fundamentals and began to form grassroots organizations focused on ridding American schools of evolution (Watts 2018). This focus on education came about as a result of the timing of Kellogg’s publication since he declared this link between war atrocities and evolution just three years after evolution was first introduced into textbooks, during a time of rapid public-school expansion. So, as American students were just beginning to learn about evolution and Darwin’s theory, their parents were learning that this theory was the root cause of German militarism, as German military and intellectual leaders had justified their imperialistic expansion using classic social Darwinism (Kellogg 1917; Blancke et al. 2014; Shermer, 2006).

In an article in *The Atlantic* in 1924, Kellogg attempted to clarify his understanding of evolution stating, “So I want to plead for a wider conception of evolution, a conception as wide as that of living Nature itself.” He also attempted to clarify his own views on the perceived conflict between evolution and Christian belief, stating, “Evolution makes its appeal to reason, but its acceptance does not mean the abasement, let alone the denial, of emotion, faith, and religion…In a word, evolution and the tenets of the Christian religion are not in opposition. They have really little to do with each other”. Yet, this publication did not dissuade the fundamentals who were already committed to preventing the perceived loss of a Christian nation to the theory of evolution as they began to lobby nationwide for legislation that would ban the teaching of evolution in public schools.

Bryan and the fundamentals were successful and evolution prohibition legislation was passed in multiple states such as Tennessee, which passed the Butler Act in 1925, which stated: “Be it enacted by the General Assembly of the State of Tennessee, That it shall be unlawful for any teacher in any of the Universities, Normals and all other public schools of the State which are supported in whole or in part by the public school funds of the State, to teach any theory that denies the story of the Divine Creation of man as taught in the Bible, and to teach instead that man has descended from a lower order of animals.” (Watts 2018).

This organized fight against the teaching of human evolution that took place around the 1920s can be seen as the official origin of the creationist movement in the USA. Although WWI ended more than one hundred years ago, its impact on how fundamentalists see science and evolution is still palpable today and can be traced back to the link that Kellogg made between Neo-Darwinism and German militarism. While the general fear of evolution has remained relatively constant, the form of anti-evolution trends today differs greatly from those of the early twentieth century. In the next section, we examine how and why creationism has evolved over the last 100 years, how it transgressed across the borders of the USA and how it has changed the public’s understanding and view of science.

## Metamorphosis of creationist thought and strategy

The creationist trend that began in the 1920s has not remained static. In fact, the creationists themselves have undergone a great transformation or evolution due to pressures in their environment. When creationism began as an organized movement, the vast majority of creationists were ‘old earth creationists.’ In the century that has passed since the beginning of the creationist movement, we have seen a rise of ‘young earth creationists’ and a new strain of creationists known as ‘Intelligent Design proponents.’

Chronologically, Old Earth Creationism (OEC) was the first major trend in the USA. As its name implies, OEC is characterized by the acceptance of the age of the earth through varying modifying the timeline of Old Testament. When the majority of creationists maintained this OE view, the attack on evolution was characterized by a direct rejection of Darwin’s concepts, seen in the passage of multiple statewide bans on the teaching of evolution (Watts et al. 2016a, b). The fundamentalists involved in this quest to stop the teaching of Darwin’s ideas clearly stated that they did not want any theory to be taught in schools that contradicted the biblical account of the creation. This anti-evolution trend was halted by a 1968 Supreme Court ruling, *Epperson v. Arkansas*, 393 U.S. 97, that stated that it was a violation of the US Constitution to prevent schools from teaching evolution (Watts 2018; Kutschera et al. 2018).

This legal loss allowed OEC to be annexed by young earth creationism (YEC)—a sect that rejects the idea of an old planet and claims that the Earth is between 6000 and 9000 years young. The trend toward YEC in the 1960s was led by Henry M. Morris (1918–2006), who was a hydraulic engineer and claimed not only that the Genesis account of creation was to be understood as literal truth, but that the truth of creation could be proven using science. Their strategy can be characterized by a promotion of creation science or scientific creationism and lobbying for legislation that would require creation science to be taught parallel to evolution in biology classes. This trend was again halted through legal action in 1987 in the Supreme Court ruling, *Edwards v. Aguillard*, 482 U.S. 578, which proclaimed that the teaching of ‘Creation Science’ was unconstitutional.

**Fig. 5:**
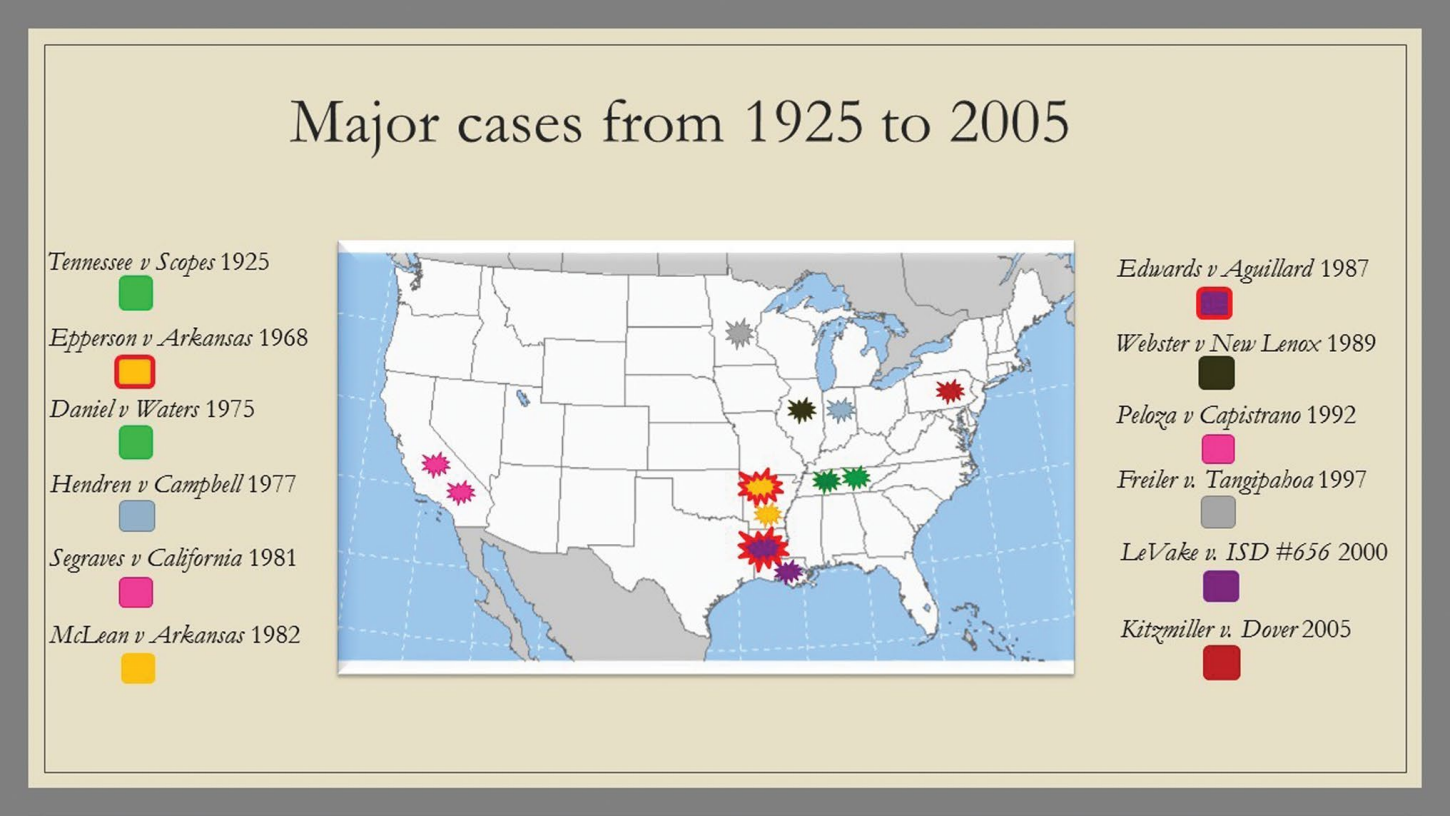
Overview of the 12 cases involving creationism (and Intelligent Design) between 1925 and 2005 in the USA

Following the 1987 ruling, Intelligent Design (ID) took center stage. This new creationist trend moved away from Genesis and focused instead on the New Testament, specifically on John 1:1: “In the beginning was the Word and the Word was God.” By doing this, ID was able to bypass all debates over Genesis and instead acted as a ‘big tent’ for creationists—drawing together Christians across a wide range of disciplines and positions, from strict YECs to theistic evolutionists (Johnson 2000). A new push for the integration of ID-principles into the classroom began, which was tested in *Kitzmiller v Dover* in 2005 and ended when the judge ruled that ID was equally as religious in nature as scientific creationism (Watts 2018; Kutschera et al. 2018). The causes for these shifting trends were related not only to the leaders’ personal beliefs and personal circumstances but largely steered by the results of legal decisions (Fig. 5).

To understand how court decisions affected and caused some of these shifts in the creationist movement, we offer a brief overview of the cases involving creationism between 1925 and 2005. Between these 80 years, there were twelve cases in a total of eight states. Each of the cases that occurred revolved around one central question: whether or not an educational practice was constitutionally valid. The absence of a legal battle in any of the other states could mean a number of different things: (1) there is little to no creationist activity in those areas, (2) there is no anti-creationist activity in those states, (3) conflicts were settled outside of court (Watts 2018).

**Fig. 6:**
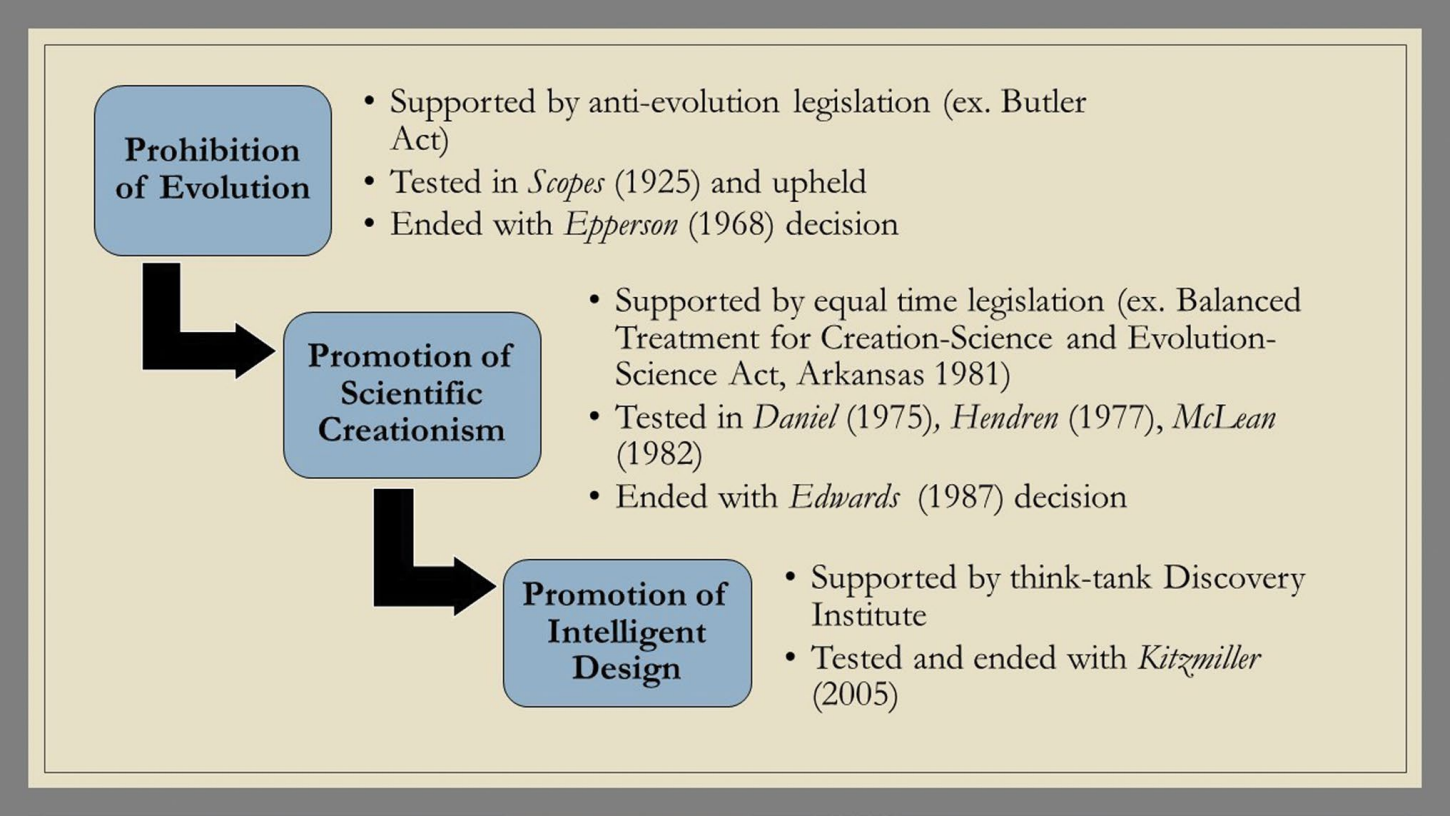
Simplified overview of the major shifts in creationism and creationist strategy due to legal rulings

So, while the combination of public-school expansion (early 1900s), the introduction of evolution into school textbooks (1914), the technological focus of warfare in WWI (1914—1918) and Kellogg’s connection between German war atrocities and Neo-Darwinism (1917) got the creationist ball rolling, once the movement began, it continued to change and transform in response to social trends and legal pressures. Figure 6 offers a simplified portrayal of how the major shifts came to be through legal decisions. The complex political, legal, societal nature of both the roots and transformation of the creationist movement highlight the fact that this creationism/evolution conflict cannot be reduced to a simple religion vs. science paradigm.

Finally, it should be mentioned that the Seattle-based *Discovery Institute*, a major “think tank” in the promotion of ID-creationism in the USA (credo: Darwinian evolution cannot construct anything new, Behe 2020), has helped to establish a “branch” in Europe. In early 2019, an Austria-based “Zentrum für Biokomplexität & NaturTeleologie” (Center for BioComplexity & Natural Teleology) published a webpage promoting ID-creationism in cooperation with the *Discovery Institute*/Center for Science & Culture (ZBKNT 2020). The new “ID-Zentrum” should be interpreted within the context of Germany’s oldest creationist association, the “Studiengemeinschaft Wort + Wissen” (Word and Knowledge Society), which recently celebrated its 40th anniversary (Schmidtgall 2019). One prominent member of W + W, the microbiologist S. Scherer (co-editor of the Bible-based *Critical Textbook*, Junker and Scherer 2013), is the second chairman of the Zentrum (ZBKNT 2020). The question as to a possible cooperation between W + W and the new *Discovery Institute*-supported ID-Zentrum, that distributes its theistic messages via YouTube, Twitter and Instagram, is open.

## Conclusions

Evidence suggests that the chain reaction that sparked the rise of this organized creationist movement was (1) the expansion of secular public schooling at the turn of the century, (2) the introduction of evolution into school public school textbooks in 1914, (3) the American’s horror of German militarism during WWI from 1914 -1918, (4) Kellogg’s link between Darwinism and German militarism, and (5) Kellogg’s subsequent influence on Bryan who then began a nationwide crusade against the teaching of evolution in American schools. And now, more than a century after the creation movement began, attempts to prevent students from learning about evolution continue today in the USA, Europe, and many other countries.

## Footnotes

1 https://www.nytimes.com/2018/11/10/science/chemical-weapons-world-war-1-armistice.html?ref=nyt-es&mcid=nyt-es&subid=article (Accessed 19 November 2018).

2 https://www.politico.com/magazine/story/2018/11/12/world-war-one-science-222410 (Accessed 20 November 2018).

3 As detailed in numerous books and articles, Weismann was the most prominent founder of what we today call the “Neo Darwinian theory” of organismic evolution (Kutschera and Niklas 2004, Kutschera and Hoßfeld 2013).
